# A wideband cryogenic microwave low-noise amplifier

**DOI:** 10.3762/bjnano.11.131

**Published:** 2020-09-30

**Authors:** Boris I Ivanov, Dmitri I Volkhin, Ilya L Novikov, Dmitri K Pitsun, Dmitri O Moskalev, Ilya A Rodionov, Evgeni Il’ichev, Aleksey G Vostretsov

**Affiliations:** 1 Novosibirsk State Technical University, K.Marx-Av.20, Novosibirsk, 630073, Russia; 2 FMN Laboratory, Bauman Moscow State Technical University, 2-nd Baumanskaya str. 5, Moscow, 105005, Russia; 3 Dukhov Automatics Research Institute, (VNIIA), 22 ul. Sushchevskaya, Moscow, Russia, 127055; 4 Leibniz Institute of Photonic Technology, PO Box 100239, D-07702 Jena, Germany

**Keywords:** cryogenic low-noise amplifier, high-electron-mobility transistor (HEMT), HEMT amplifier, microwave cryogenic amplifier, microwave superconducting circuit readout, superconducting qubit readout

## Abstract

A broadband low-noise four-stage high-electron-mobility transistor amplifier was designed and characterized in a cryogen-free dilution refrigerator at the 3.8 K temperature stage. The obtained power dissipation of the amplifier is below 20 mW. In the frequency range from 6 to 12 GHz its gain exceeds 30 dB. The equivalent noise temperature of the amplifier is below 6 K for the presented frequency range. The amplifier is applicable for any type of cryogenic microwave measurements. As an example we demonstrate here the characterization of the superconducting X-mon qubit coupled to an on-chip coplanar waveguide resonator.

## Introduction

Quantum microwave devices are widely used for different applications ranging from radio astronomy [1–3] to quantum information processing circuits [4]. The latter include the most challenging and attractive topics such as quantum bits (qubits) [5], quantum dots [6], microwave single-photon detectors [7], high-quality resonators [8], superconducting microwave beam splitters [9], and other circuit quantum electrodynamics structures aimed to be used as elements for quantum processors. The experimental study of the abovementioned quantum devices requires precise low-noise readout electronics including low-noise amplifiers [10].

Two main low-noise amplifiers parameters are small equivalent noise temperature (*T*_n_) and relatively high gain (*G*). The superconducting qubit measurement setup requires particular low-noise amplifiers applicable for cryogenic temperatures. An important point is that cryogenic low-noise amplifiers (cLNAs) are wideband noise sources itself. In the superconducting qubit experimental setup the noise generated from a cLNA is introduced to the sample, which might be crucial for the main qubit parameters, especially for the relaxation time *T*_1_ and coherence time *T*_2_. Therefore, cryogenic microwave isolators are used between the sample and the cLNA.

Most of the modern cryogenic amplifiers operate at temperatures of liquid helium [11–16] and are, usually, placed at the 4 K stage of dilution refrigerators. These amplifiers can be implemented using two modern semiconductor transistor technologies, that is, high-electron-mobility transistor (HEMT) technology, including GaAs and InP, and SiGe heterojunction bipolar technology (HBT). Recently, it has been shown that cryogenic amplifiers based on SiGe HBT [16–18] can provide low noise levels and high gain at 4 K. They are suitable for superconducting qubit readout [25–26] and radio astronomy systems [12,14].

In order to obtain high gain values for *C* (ranging from 4 to 8 GHz) and *X* (ranging from 8 to 12 GHz) frequency bands the number of amplification stages of the HBT cLNA should be increased. This leads to an increase of the total power dissipation in the dilution refrigerator, which makes its operation unstable. In contrast, using modern HEMT technology allows one to implement cryogenic amplifiers with high gain values for a wide frequency range up to tens of gigahertz [11,13,19]. Most of them are based on monolithic microwave integrated circuit (MMIC) technology, which yields outstanding noise parameters in the *C* and *X* frequency bands. However, for some applications a flexible amplifier design is needed. Indeed, there are known schematic realizations of cLNAs based on discrete components [11,17,20–24]. The experimental characteristics of the presented amplifiers show that they do not provide gain values of more than 30 dB in the frequency range from 8 to 12 GHz. The evolving semiconductor technology provides the modern market with state-of-art commercially available transistors to substitute old types. A new type of commercially available transistors was used for the cLNA presented in this paper. A realization of a microwave frequency cLNA design, based on commercially available HEMT transistors, requires accurate and precise matching and an appropriate selection of passive components for liquid-helium temperatures.

In this paper we show that an accurate microwave matching circuit design based on commercially available transistors yields low-cost stable cryogenic low-noise amplifiers with a frequency range up to 12 GHz. The implemented cLNA has the following parameters: a gain value of more than 30 dB for a frequency range from 6 to 12 GHz and an equivalent noise temperature below 6 K. The cLNA has 50 Ω input and output terminations and can be installed in a microwave measurement setup at the 4 K stage of modern dilution refrigerators. The implemented amplifier was tested by superconducting X-mon qubit measurements. In order to measure the X-mon qubit parameters the standard low signal power one microwave tone [25–26] and two microwave tone [27] spectroscopy experiments were performed.

## Results and Discussion

### Cryogenic four-stage low-noise amplifier design

The amplifier design was optimized for low noise, adequate gain, and appropriate output matching at cryogenic temperatures. The latter is important for better matching with room-temperature amplifiers. CE3512K2 GaAs pseudomorphic HEMT transistors made by CEL were used. The transistors were selected with regard to the two following parameters: minimum noise figure (NF) of 0.3 dB and minimum required value of associated gain of 12 dB at a frequency of 12 GHz. The design was optimized for the frequency range from 6 to 12 GHz, which fits to most of modern qubit measurement setups. We used the available S-parameters of the transistor for the matching circuit design. From a circuit simulation with ideal components a minimal gain value of more than 36 dB at 300 K in the frequency range from 6 to 12 GHz was obtained. For the next step we used the real S-parameters of passive circuit components placed on a dielectric substrate. High-frequency edged trimmed block resistors with a 0402 package size from Vishay company were used in drain and gate biasing lines. The appropriate selection of the resistors in cryogenics is a crucial point in amplifier implementation. We should mention that the first stage of the amplifier does not have a gate resistor in its circuit. One of the reasons is a noise reduction, which is introduced to the input circuit of the amplifier. For increasing the circuit stability around 10 GHz and better matching, 10 Ω gate resistors were used for the second and the fourth stage. Multilayer ceramic capacitors with C0G dielectric material in a 0402 package were used. Murata ceramic core coils with 0201 SMD package size and a self-resonance frequency of about 20 GHz were used for gate and drain power supply circuits. All passive components, for example, microwave capacitors, coils, and precision resistors were measured before at liquid helium temperature for obtaining the spread of nominal parameters. The amplifier was assembled on a Rogers RO 4350 substrate and the matching circuits were carried out as microstrip lines.

**Figure 1:**
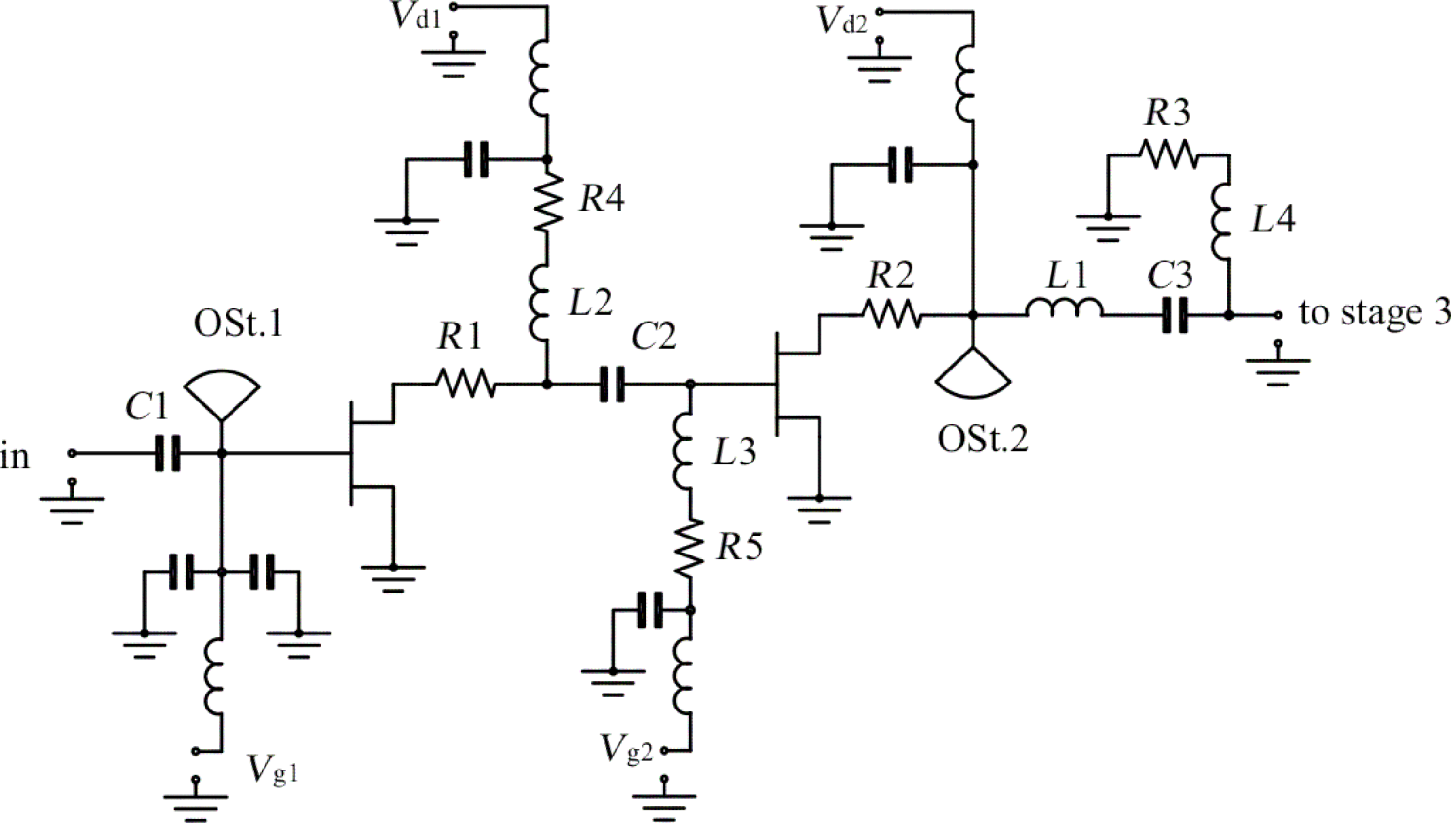
Schematic of the 6–12 GHz cryogenic LNA. The important component values are: *C*_1_ = 0.6 pF, *C*_2_ = 0.3 pF, *C*_3_ = 0.2 pF, *L*_1_ = 0.8 nH, *L*_2_ = 0.8 nH, *L*_3_ = 0.6 nH, *L*_4_ = 3 nH, *R*_1_ = 10 Ω, *R*_2_ = 18 Ω, *R*_3_ = 25 Ω, *R*_4_ = 25 Ω, *R*_5_ = 10 Ω. The capacitors and resistors are realized in 0402 packages and the inductances are realized in a 0201 package (OSt.: open stub lines). The other passive components do not affect to the signal matching and are used for filtering of the bias lines.

**Figure 2:**
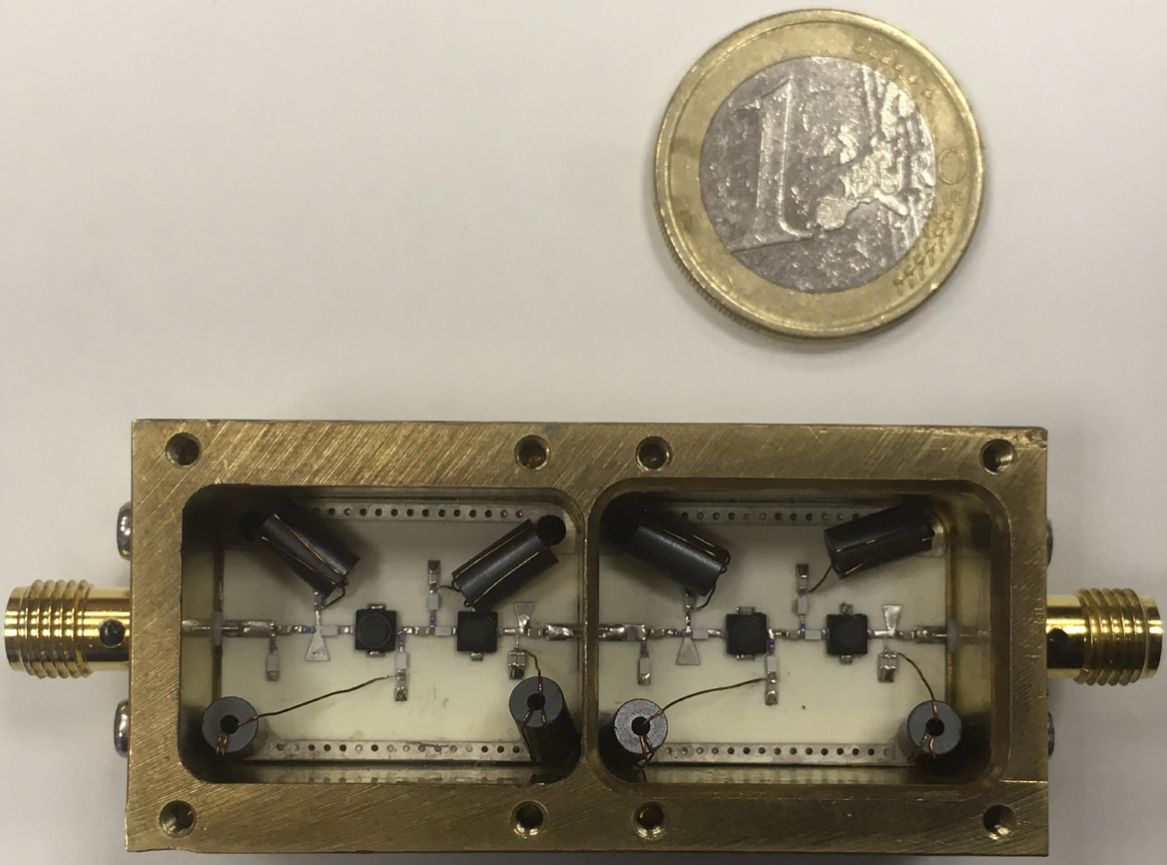
Realization of the cryogenic LNA.

The amplifier was mounted inside a brass box separated into two chambers. Each chamber contains two stages of the amplifier. The separation was made in order to avoid standing waves and self-resonances in the box in the operation frequency range. We did an electromagnetic simulation of the box and it showed us the absence of self-resonances in a frequency range from 1 to 12 GHz. The substrate was soldered directly to the brass box and SubMiniature version A (SMA) connectors with a specified frequency range up to 12 GHz were soldered directly to the microwave laminate. The feedthrough filters were used as the input biasing electrodes and the power supply cables from a micro-D cryostat connector were soldered directly to them. The first two stages of the amplifier circuit are presented in Figure 1. The third and the fourth stage are identical. One of the key elements in the matching circuits is an open stub line (marked as OSt. in Figure 1) in the gate circuit of the input stage and in the drain circuit of the second and the fourth stage (Figure 2).

### Cryogenic LNA characterization

**Figure 3:**
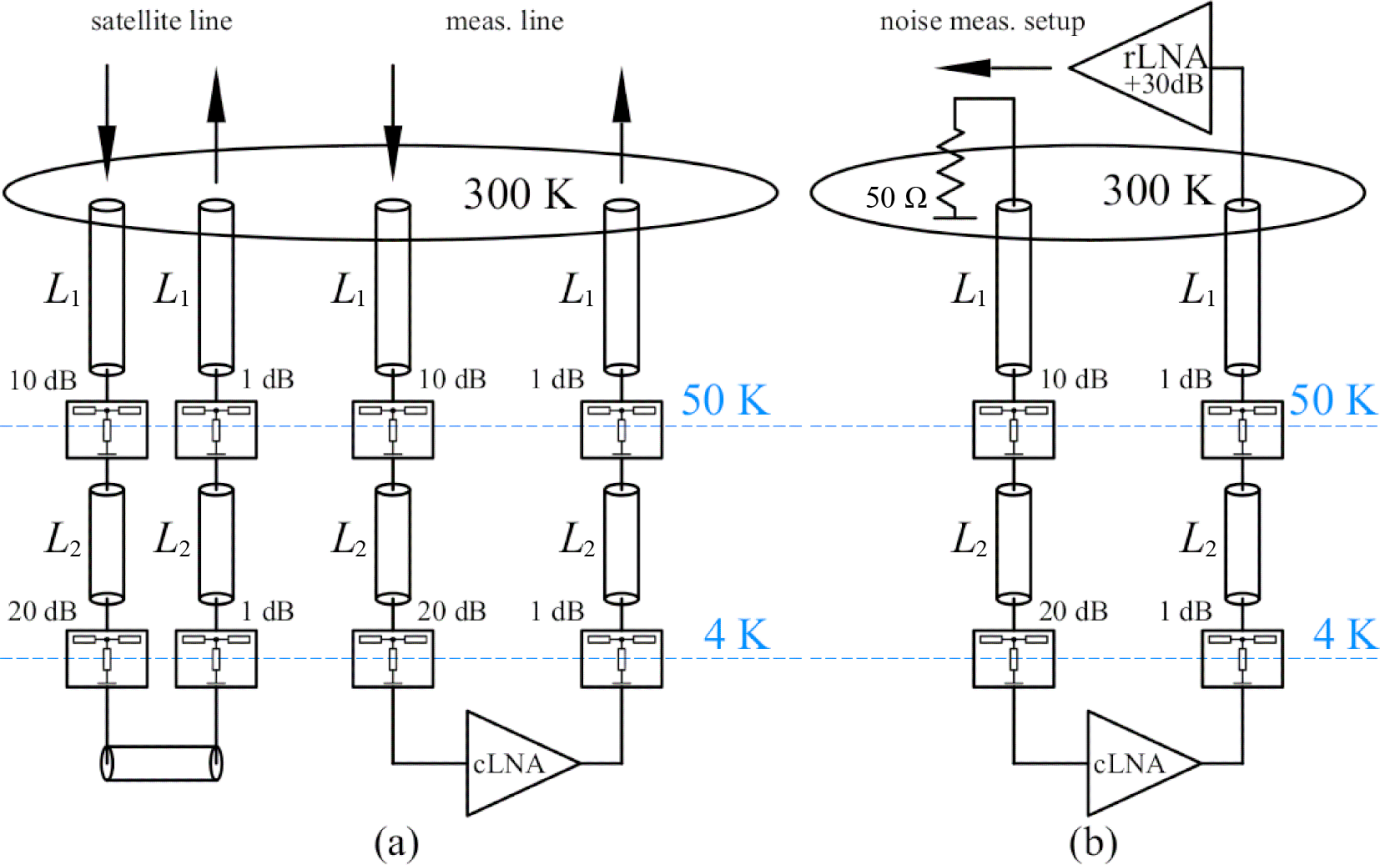
Noise and gain measurement setup. (a) Calibration lines (left-hand side sketch) and gain measurement setup (right-hand side sketch). (b) Noise measurement setup for the cryogenic LNA.

The measurement system of the amplifier was based on a 10 mK closed-cycle dilution refrigerator where the amplifier was installed and fixed with screws to the copper holder at the 4 K plate. We used standard 2.2 mm CuBe 50 Ω coaxial microwave lines between 4 K stage and room-temperature SMA flange as input and output signal lines installed into the dilution refrigerator for the S-parameter and noise temperature measurements of the amplifier. The simplified measurement scheme is presented in Figure 3. In order to reduce the input signal level, to perform thermal anchoring at each stage, and to prepare the noise measurement setup, cryogenic broadband 10 dB and 20 dB attenuators were installed at the 50 K flange and the 4 K flange, respectively. Moreover, with the described setup we were able to perform gain and noise measurements during one cooldown without rebuilding the measurement scheme and warming up the cryostat. An additional channel with identical microwave coaxial cables and cryogenic attenuators was used for compensating the losses and calibrating the noise temperature. It is marked as satellite line in Figure 3. A short 2.2 mm 50 Ω coaxial cable with SMA termination was used to connect the satellite lines. Gain and noise properties of the amplifier were characterized at the exact temperature of 3.8 K.

**Figure 4:**
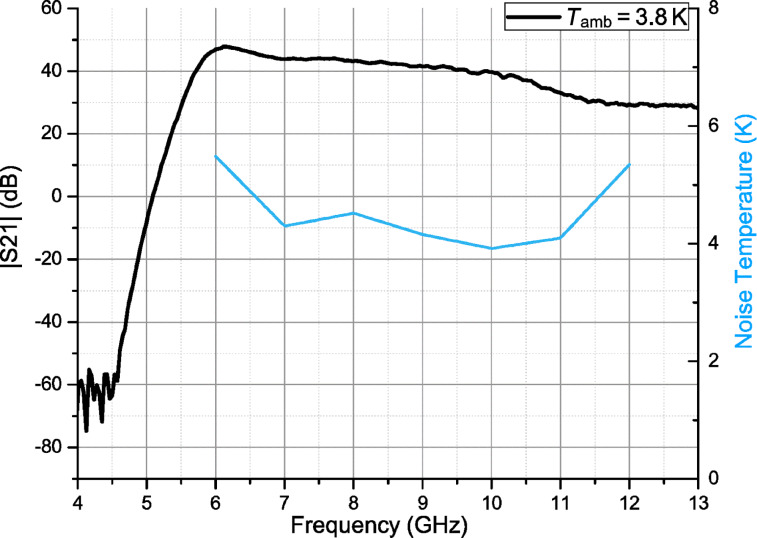
Gain and noise temperature of the cryogenic LNA at the experimental temperature of 3.8 K.

We designed and implemented an independent eight-channel power supply unit based on Pb batteries, which allowed us to bias drain and gate circuits of each stage independently. The S-parameters of the designed cLNA and the satellite lines were measured using a vector network analyzer (VNA) in a frequency range from 4 to 13 GHz and with an output signal power level of −20 dBm. The total power applied to the input of the cLNA was lower than −50 dBm. We performed transmission measurements of the satellite lines and calibrated our measurement setup according to the loss rate. The gain curve is presented in Figure 4. We were not able to measure the reflection coefficients during the cooldown because of the setup features. The output reflection of the cLNA |*S*_22_| was measured separately and the reflection level did not exceed −10 dBm for the presented frequency range.

The noise measurement procedure required an approach with higher precision. In this work we estimated the highest equivalent noise temperature level. We used the VNA in the spectrum analyzer mode and measured the power noise density at the output of the setup. In order to increase the sensitivity we applied an additional low-noise broadband room-temperature microwave amplifier (see rLNA in Figure 3a) directly to the 300 K cryostat SMA output flange. The gain *G*_2_ and noise *P*_2_ properties of the rLNA were characterized before by means of a calibrated noise source and the Y-factor procedure [28]. In order to terminate the input line we placed a broadband 50 Ω load at the cryostat microwave input at the 300 K flange SMA connector, see Figure 3b. Using the setup shown in Figure 3b we measured the total noise power density *P*_total_ in the frequency range from 6 to 12 GHz. It is defined as

[1] $$P_{\text{total}}=P_{1}t+P_{2}G_{2}+P_{\text{VNA}},$$

where *P*_1_ is the cLNA input power noise density, *P*_2_*G*_2_ is the output power noise density of the room-temperature low-noise amplifier, *P*_VNA_ is the spectrum analyzer self-noise, and *t* is the transmission coefficient from the input of the cLNA to the output of the rLNA. It is defined as

[2] $$t=\frac{G_{1}G_{2}}{L_{\text{out}}},$$

where *L*_out_ is the loss rate of the output line calibrated previously. All variables are in the linear scale. The output line loss coefficient depends linearly on the frequency. First, we obtained the power noise density of the cryogenic LNA with an exception of rLNA noise and VNA noise, *P*_1_*t* = *P*_total_ − *P*_2_*G*_2_ − *P*_VNA_. Then the power noise density related to the input, *P*_1_, was obtained and the equivalent noise temperature was calculated according to the formula

[3] $$T_{\text{Na}}=\frac{P_{1}}{k_{\text{B}}}.$$

The noise curve is presented in Figure 4 and the equivalent noise temperature does not exceed 6 K for the frequency range from 6 to 12 GHz. The minimum noise temperature of 4 K was obtained at 10 GHz. This can be explained by better noise matching at 4 K where the parameters of the passive components are slightly different. The amplifier parameters measured at 3.8 K are summarized in Table 1. The amplifier exhibits low equivalent noise temperature and high gain. Therefore, it is suitable for the superconducting qubit readout.

**Table 1:** The main parameters of the cryogenic LNA.

*G*_min_	BW	max *T*_noise_	*P*_cons_	*T*_amb_
[dB]	[GHz]	[K]	[mW]	[K]

30	6–12	6	20	3.8

The main parameters in Table 1 were obtained for the optimum working points for each amplifier stage with the following operating drain currents: *I*_d1_ = *I*_d2_ = 3.3 mA, *I*_d3_ = 5.8 mA, *I*_d4_ = 6 mA. Increasing the drain working currents to 10 mA does not lead to an increase of gain but significantly increases the noise temperature to 15 K with a total power consumption of 73 mW. It is important to note that during the cLNA and qubit measurement experiments the dilution refrigerator was working stable with a constant He_3_/He_4_ mixture flow rate and with stable temperature values at each cooling stage.

### X-mon qubit characterization

The implemented cryogenic LNA is dedicated for measurements in a frequency range from 6 to 12 GHz and it was installed at the 4 K stage of the dilution refrigerator. The sensitivity and measurement speed of the full measurement setup is obviously defined by the signal-to-noise ratio and, thus, by the gain and the noise temperature of the cLNA.

The sample under study contained superconducting X-mon qubits coupled to coplanar quarter wavelength resonators. Each resonator was coupled to a single qubit. The sample was made at the BMSTU Nanofabrication Facility (FMN Laboratory, FMNS REC, ID 74300) using Al technology [29–30]. The X-mon qubit was made of two Al/Al*_x_*O*_y_*/Al parallel Josephson junctions forming a SQUID-type structure coupled to the main ground plane. The standard spectroscopy experiments were carried out using low input signal powers.

**Figure 5:**
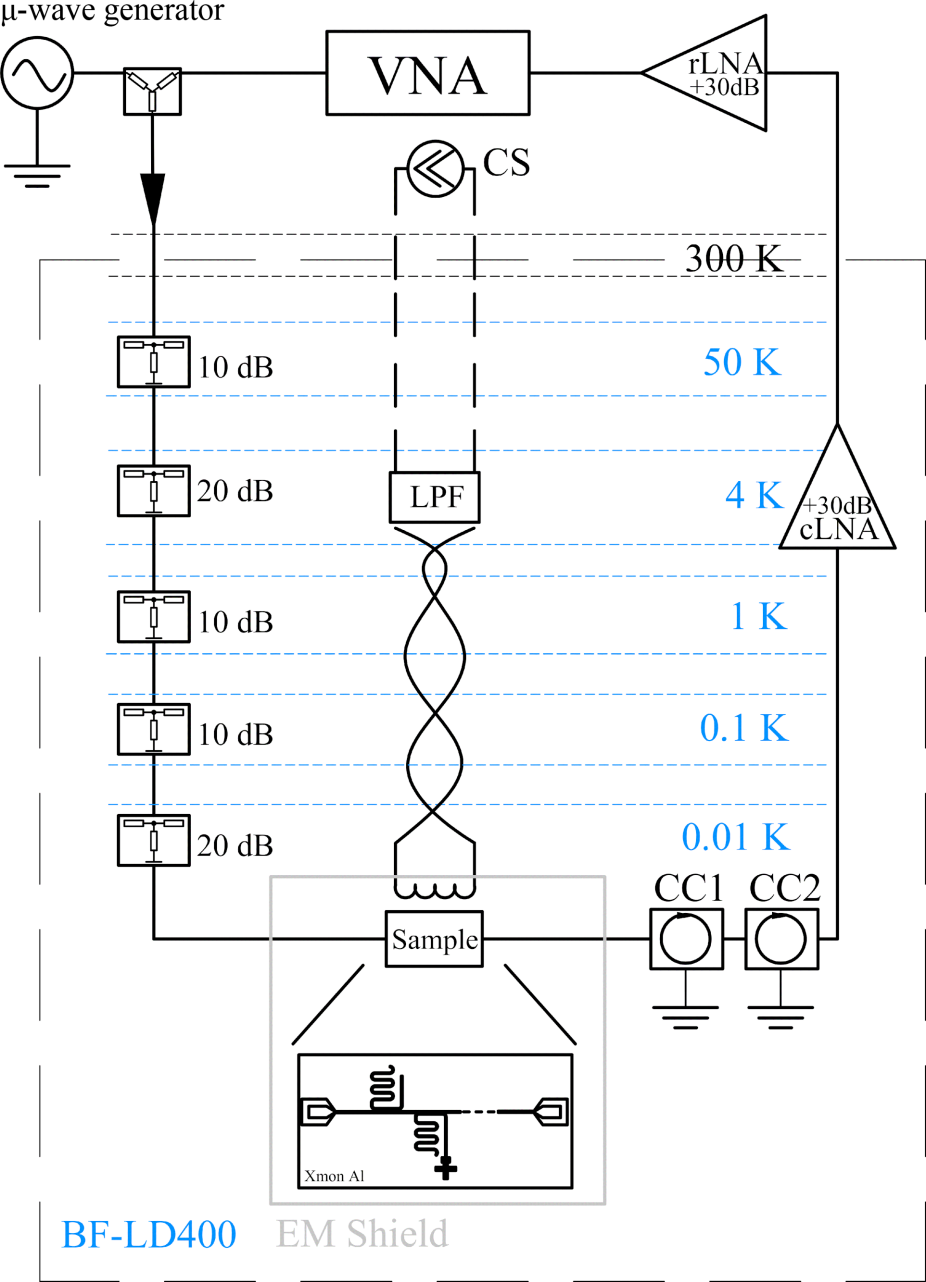
The X-mon qubit measurement setup. The implemented amplifier is marked as cLNA and the room-temperature amplifier is marked as rLNA. CC1 and CC2 are cryogenic circulators with 50 Ω termination (LPF: low-pass filter for the superconducting coil line, CS: high-precision current source). The following setup was installed in a BlueFors BF-LD400 dilution refrigerator.

The qubit measurement setup is presented in Figure 5 where the designed and implemented amplifier is marked as cLNA. In order to decouple the sample from external noise sources we used two cryogenic circulators (marked as CC1 and CC2 in Figure 3) installed in series at the output of the sample and wideband cryogenic attenuators installed in a microwave input line, see Figure 5. The two circulators placed in series at the 10 mK stage had a direct loss rate of about 4 dB and a return loss rate of about 40 dB in our measurement frequency range. Their main function was to isolate the sample from cLNA noise.

Wideband cryogenic attenuators (marked as 10 dB and 20 dB) for room-temperature noise suppression and noise suppression of each next following temperature stage were placed according to the scheme in Figure 5. The VNA and a wideband stable microwave sweeping signal generator (marked as “μ-wave” generator in Figure 5) were used for spectroscopy measurements. The standard heterodyne measurement method was used. A superconducting Nb coil was used for tuning the qubit eigenfrequency for each experiment. The coil was biased by means of a highly stable current source (marked as CS in Figure 5). The sample was placed in the copper sample holder with Pb and Mu-metal electromagnetic shields at the 10 mK temperature stage. A two-stage differential low-pass filter (marked as LPF) with 10 kHz bandwidth and wide stopband frequency range was implemented characterized and installed into the measurement system at the 4 K temperature stage of the diluton refrigerator. Since the power level of the measured signal applied to the sample was approximately −120 dBm and the gain of the cryogenic LNA was 30 dB, an additional room-temperature low-noise microwave amplifier (marked as rLNA in Figure 5) was used. It was dedicated to increasing the signal level at the output of cLNA before data acquisition. The rLNA did not influence the signal-to-noise ratio of the measurement system since this parameter is mainly defined by the cLNA output noise. The rLNA noise figure corresponded to 2.1 dB with a gain value of about 40 dB for the frequency range from 6 to 13 GHz.

**Figure 6:**
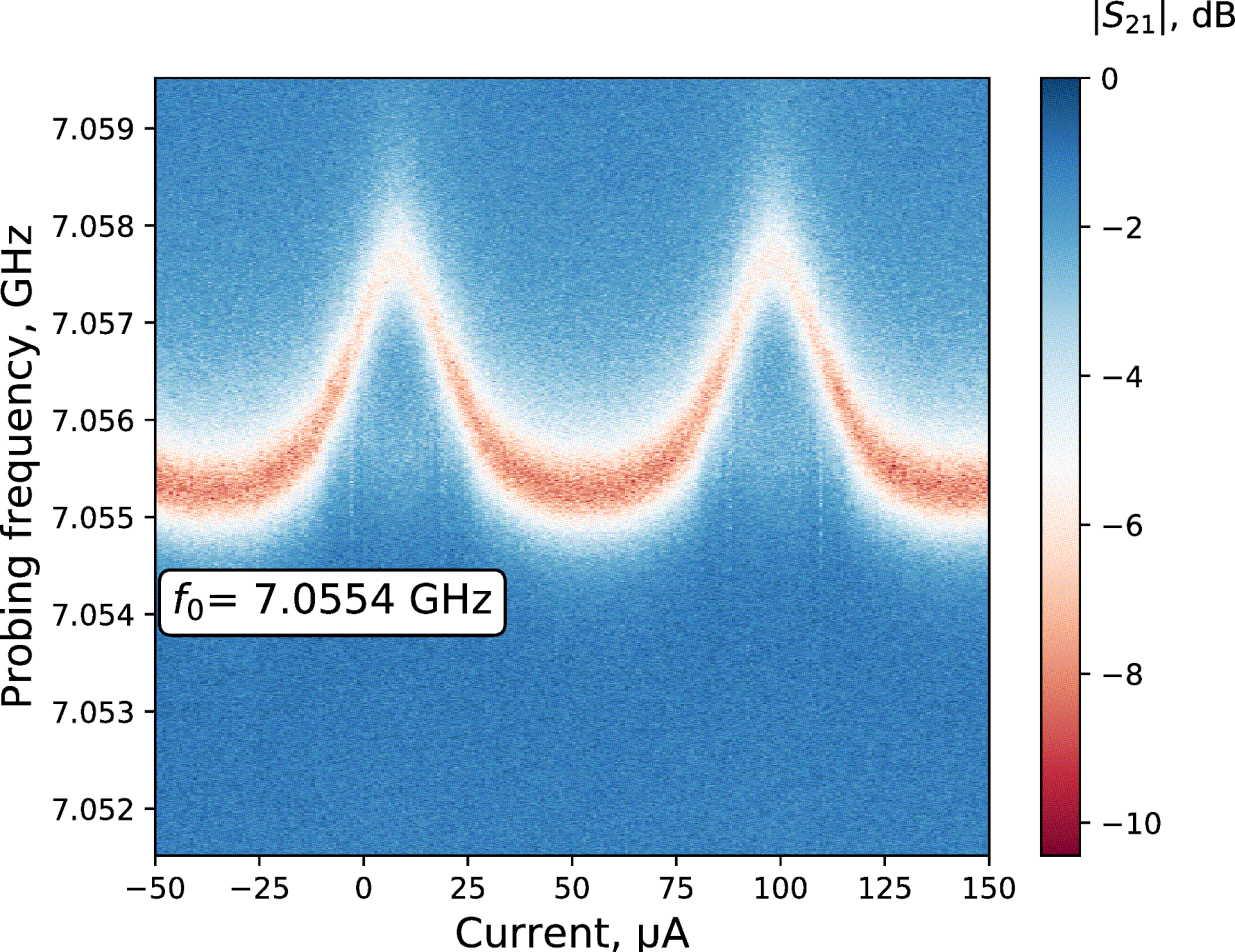
The qubit “sweet spot”. The transmission (color intensity graph with the blue color corresponding to the maximum and the red color corresponding to the minimum) at the resonator resonance frequency depending on the external flux bias for the frequency range from 7.0515 to 7.0595 GHz (vertical axis) and a constant probing power of −120 dBm.

The transmission |*S*_21_| of the sample was measured and the resonance frequencies of resonators were defined in the frequency spectrum from 6 to 9 GHz. We defined a resonator fundamental frequency of *f*_0_ = 7.0554 GHz and experimentally characterized the qubit responce. First, one-tone spectroscopy was carried out. The transmission of a sweeping microwave signal through the sample in a frequency range of 7.05–7.06 GHz for different DC bias currents, producing an external magnetic field, was measured. The obtained curve is shown in Figure 6.

Here, the horizontal axis is a sweeping DC magnetic field in current values, the vertical axis is the sweeping frequency of a probing microwave signal and the color intensity graph shows the normalized transmission amplitude |*S*_21_| in units of dB, where 0 corresponds to a normalized maximum transmission. Due to the magnetic flux quantization the qubit characteristic has periodic behavior. The maximum shift of the resonance frequency corresponds to a so called “sweet spot” and the distance between two sweet spots corresponds to the magnetic flux quantum Φ_0_. We would like to mention that the current graph was obtained with 100 Hz of the intermediate frequency VNA filter bandwidth and for our experimental setup only four averages were required. In this sense, the cLNA reduces the measurement time of the experiment, which is especially important for preliminary qubit experiments.

**Figure 7:**
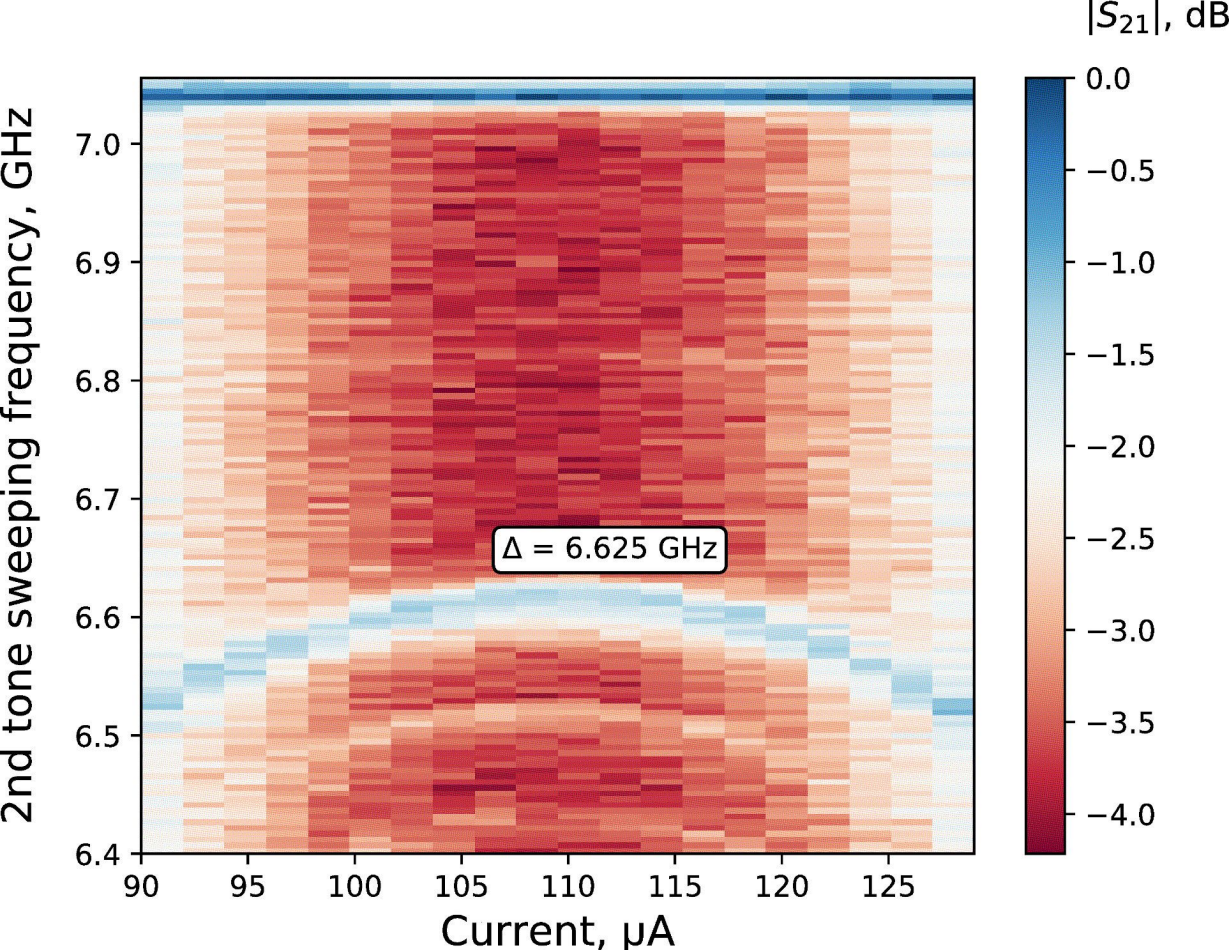
The qubit spectroscopy. The transmission dependence of the probing signal (color intensity graph) on the bias current (horizontal axes) for different values of driving signal frequency in a range from 6.4 to 7.1 GHz (vertical axes).

We have performed also a standard two-tone spectroscopy measurement using an additional stable wideband microwave generator, marked as “μ-wave generator” in in Figure 5 and a resistive power combiner. We applied two microwave tones, that is, first a probing signal tone and then sweeping driving signal tones in a frequency range of 6.4–7.1 GHz. The powers applied to the sample after the input line attenuation were −120 dBm for the probing signal tone and −80 dBm for the driving signal tone, respectively. We defined the qubit spectrum depending on the external flux bias and found a minimum qubit energy gap of Δ = 6.625 GHz. The qubit spectrum as a function of the current is shown in Figure 7. Here, the transition |*g*⟩–|*e*⟩ is shown.

## Conclusion

The characteristics of a low-noise cryogenic microwave amplifier and the measurement of a superconducting X-mon qubit are shown. The amplifier has a gain of more than 30 dB and a noise temperature lower than 6 K and is suitable for any high-sensitivity experiments in the presented frequency range. It is also necessary to mention that, during the qubit measurements with the implemented cLNA, the cryostat operating parameters, that is, the temperature of the stages and the cryostat He_3_/He_4_ mixture flow rate, were stable. The two-tone qubit spectroscopy experiment shown in this paper required about 48 h of continued measurements with the operating amplifier. The spectrum does not have any artifacts and shows a defined dependence on the external flux. Therefore, we conclude that the main parameters of the amplifier were stable during this time. From the noise curve it is seen that it is possible to achieve a noise temperature of 4 K. This fact can be explained by a better noise matching for this particular cryogenic temperature.
